# Children’s Academic, Artistic, and Athletic Competencies: Successes Are in the Eye of the Beholder

**DOI:** 10.3389/fpsyg.2019.02225

**Published:** 2019-10-22

**Authors:** Sarah J. Racz, Diane L. Putnick, Gianluca Esposito, Marc H. Bornstein

**Affiliations:** ^1^University of Maryland, College Park, College Park, MD, United States; ^2^Eunice Kennedy Shriver National Institute of Child Health and Human Development, Bethesda, MD, United States; ^3^Nanyang Technological University, Singapore, Singapore; ^4^University of Trento, Trento, Italy; ^5^Institute for Fiscal Studies, London, United Kingdom

**Keywords:** academic competencies, mean differences, rank order agreement, perceptions of academic performance, teacher perspectives

## Abstract

A significant challenge to fully understanding children’s academic and other competencies is dependency of the determination on the method of study, including notably who makes the assessment. This study examined similarities and differences in child, mother, father, and teacher reports of children’s competencies across multiple domains of math, reading, music, and sports from two separate perspectives of rater agreement, mean level and order association. Two hundred and sixty-seven European American families were recruited from the mid-Atlantic region of the United States, and children, mothers and fathers, and teachers completed a commonly used rating measure of children’s competencies when the children were 10 years of age. Results showed (1) high levels of order agreement (perhaps reflecting the observable nature of children’s competencies), (2) some systematic mean level differences across raters, and (3) little inter-domain agreement (except among teachers, which may reflect teachers’ unique perspectives on children’s competencies). The educational, developmental, and methodological implications of the findings are discussed in the context of children’s school performance. Who makes the determination of children’s several different competencies matters.

## Introduction

Individual perceptions of children’s academic and other competencies have important implications for their scholastic achievements and classroom adjustment as well as their overall feelings of self-worth (Wigfield et al., 1991; McGrath and Repetti, 2000; Wentzel et al., 2016). Importantly, systematic differences in reports of children’s competencies in the school setting and elsewhere may create conflicts, biases, and expectations regarding children’s academic and other abilities (Rosenthal, 1994). Prior research suggests that these perceptions tend to become self-fulfilling prophecies, such that children perceived as more or less competent “live up or down” to those expectations and subsequently perform well or poorly in those domains (i.e., the “Pygmalion Effect”; Jussim and Harber, 2005). What is not clear, however, is whether children’s performance is due to actual individual differences in their abilities or to variability in how different observers judge their scholastic and other competencies (i.e., inter-rater agreement) or to how their competences are evaluated across different domains of performance (inter-domain agreement). Few studies have examined multi-reporter similarities and differences in perceptions of children’s performance in multiple domains commonly experienced during elementary school (i.e., math, reading, music, and sports). One aim of the current study is to address this gap in the literature.

Consider academics. Understanding how different reporters rate children’s scholastic competencies across different domains is important as it may help identify areas where there is a mismatch between expectations for how a student will perform academically. That is, disagreement in reporters’ ratings of children’s academic competences may lead to conflict or other difficulties in navigating educational settings. For instance, if a parent perceives a child’s academic competency to be higher than what the child’s teacher perceives it to be, the parent may consider any poor grades or academic difficulties to reflect unfair or biased perceptions of the child’s teacher. Often this conflict is based around interpersonal differences in educational values or expectations, and understanding how perceptions of academic competencies may contribute to such conflict may help identify ways in which to strengthen or remediate key parent–teacher learning partnerships to better support children’s academic success (Crozier, 1999; Creech and Hallam, 2003). Similarly, differences in perceptions of children’s competencies across various academic domains may help identify areas where children may need more support, or where their competencies are less readily observable to multiple reporters. For areas where children’s competency may be less observable, it may be important for teachers, parents, and children to communicate openly and regularly (e.g., parent–teacher conferences, home-school notes). Awareness of differences and similarities in these inter-rater and inter-domain perceptions may help reduce potential interpersonal conflicts and enhance the ability of parents, children, and teachers to work together to best support children’s academic progress. Thus, a second aim of the current study was to provide concrete information on agreement of children, parents, and teachers about multiple domains of children’s competencies.

Several theoretical frameworks have been advanced to understand children’s academic competencies. For instance, differences in children’s competencies across reporters and academic domains have been conceptualized within dimensional comparison theory (Möller and Marsh, 2013; Marsh et al., 2014), whereby ratings of competencies in one domain are determined, in part, by how achievement in that domain is perceived compared to achievements in other areas. Ratings of children’s academic competencies may also reflect both internal (comparing children’s competencies in one domain with their competencies in another domain) and external (comparing the child’s competencies in one domain to the competencies demonstrated by other children in that domain) processes (Marsh, 1986). More salient to the current study is Expectancy-Value Theory (EVT; Wigfield and Eccles, 2000), which asserts that expectations and values regarding achievement directly influence academic choices, performance, effort, and persistence. Such expectancies and values are influenced by various social and cognitive factors including beliefs about general abilities and perceived difficulty of tasks, which in turn are influenced by individuals’ perceptions of their own academic experiences as well as perceptions from others (e.g., parents and teachers; socialization influences). In many ways this theory proposes a mediational model of academic achievement, whereby perceptions of academic competencies (both by the self and others) lead to social and cognitive factors which lead in turn to academic expectations and values which then lead to academic achievement outcomes. Much research has documented links among expectations, values, and outcomes (e.g., Eccles and Wigfield, 1995; Wigfield et al., 1997; Simpkins et al., 2012), but less work has explored the potential role of academic perceptions of the self and other socializers. As noted above, these self and other perceptions may be particularly important to supporting student successes in an educational setting. An essential step in highlighting how these factors fit within the broader EVT model is to understand reporting patterns in perceptions of children’s academic competencies across individuals and domains. This understanding constituted a third aim of the current study.

### Ratings of Academic Competencies Across Reporters and Domains

When considering individual differences in ratings of children’s academic competencies, it is important to acknowledge that findings documenting any unique perspectives show that the degree of inter-rater disagreement often depends on the reporters being compared. Specifically, agreement between mothers’ and fathers’ ratings tends to be higher than that between other reporters (e.g., parents versus teachers), which may be due in part to the fact that mothers and fathers tend to observe children in the same contexts (i.e., home; Kerr et al., 2007; Schroeder et al., 2010; De Los Reyes et al., 2015). Between different settings (i.e., school versus home), teachers tend to report significantly fewer child difficulties as compared to parents and children (Salbach-Andrae et al., 2009). Given that teachers are exposed to a range of students, the abilities exhibited by any one student may not seem as noteworthy to teachers as they do to parents, who tend to view their children’s capabilities in more isolated and individualized settings. Teachers may therefore have a unique perspective on children’s academic competencies due to their extensive interactions with children in academic settings.

In addition to inter-reporter differences, the informant discrepancy literature indicates that ratings of children’s functioning tend to vary depending on whether the domain being assessed is easily observable or not (De Los Reyes et al., 2015). It may therefore be the case that greater inter-rater agreement is observed within academic domains that are easy to track and observe through homework assignments and test scores (e.g., math and reading) as compared to those areas with less observable and quantifiable outcomes (e.g., music and sports). Furthermore, differences in reporters’ ratings often reveal meaningful information about children’s behavior in various contexts (e.g., home versus school) and domains (e.g., internal versus external behaviors), and therefore have important implications for the identification of difficulties in children’s cognitive, academic, social, emotional, and behavioral competencies (De Los Reyes, 2011; De Los Reyes et al., 2013). For instance, children’s academic functioning may only become evident in the context of the cognitive and behavioral demands of core academic classrooms (Rimm-Kaufman et al., 2001), further highlighting the importance of comparing ratings of children’s academic competence across reporters and domains. Multi-informant assessment approaches therefore constitute a promising avenue to fully evaluate children’s competencies across both raters and domains. Overall, extensive research findings indicate that differences in reports of children’s functioning tend to vary depending on both the raters and domains assessed. The question of which raters and which domains are so influenced is only answered by a multi-informant multi-domain design with the same children as we use here.

Differential associations among various raters’ reports of children’s academic competencies, for example, underscore the importance of considering differences in perceptions of children’s competencies across reporters and domains. Specifically, children’s self-perceptions of their academic competencies have been reported to be more strongly associated with their mothers’ than their teachers’ perceptions of their academic competencies (Wigfield et al., 1997). However, teachers’ ratings of children’s literacy and math competencies in early elementary school predict children’s perceptions of their literacy and math competencies, respectively, in later elementary school, whereas only parents’ judgments of children’s math competence predict children’s later perceptions of their math competence (Herbert and Stipek, 2005). These differences in teachers’ perceptions of children’s academic competencies may be due to several factors noted above, including teachers’ unique perspectives in the classroom, the availability of official examination standards which may inform their perceptions, and the potential for their expectations to create a self-fulfilling prophecy for their students. More attention to these differential associations in reports of children’s academic competencies may identify additional explanatory factors.

Of note, most previous studies have investigated perceptions of children’s academic competencies when children are in the early elementary school years (i.e., Kindergarten and 1st grade; Wigfield et al., 1997; Herbert and Stipek, 2005). It is therefore not known if these findings extend to the later elementary school years, which represent a crucial educational transition point as children prepare to enter middle/secondary school. The current study therefore provides a developmental extension of the extant literature by examining these associations when children are in the later elementary school years (i.e., approximately 5th grade). The current study also extends findings regarding agreement in raters’ reports of children’s academic and other competencies to incorporate perspectives of children’s competencies from *four* reporters (i.e., children, mothers, fathers, and teachers) and across *four* academic domains (i.e., math, reading, music, and sports) commonly experienced in the school setting.

Furthermore, the current study provides a methodological contribution to the study of individual differences in perceptions of children’s competencies. Reporter agreement and disagreement can be evaluated using (at least) two different approaches to analysis – mean level and rank order. *Mean level* analyses assess similarities and differences in ratings for the whole group. For example, if children tend to view themselves as more competent in a given domain than parents view them, we would find a significant mean level difference between raters. Rank order analyses assess similarities and differences in correlations between raters. For example, agreement would be considered strong if two raters assess one individual’s math competence high and another individual’s math competence low relative to others in a group. Agreement would be less strong if one rater ranks individuals’ competence relatively high and another rater ranks competence relatively low in the group (and vice versa). Clearly, the two analytical approaches provide different sorts of information about rater agreement. In the current study, we assess both approaches to analysis to provide a more complete picture of inter-rater and inter-domain judgments of children’s competencies.

### Overview of the Current Study

The current study examines child, mother, father, and teacher ratings of children’s competencies in four domains commonly experienced in the school setting (i.e., math, reading, music, and sports). Our methodological approach allowed us to disentangle rater agreement that is general (e.g., similar mother-father agreement holds across all domains) from rater agreement that is specific to particular domains (e.g., raters agree about competence in music but not sports). Likewise, we disentangle rating patterns that are general to all raters (e.g., correlations between math and reading competence are similar for all raters) from rating patterns that are specific to a particular rater (e.g., child-rated competence in all domains is correlated, but mother-rated competence differs across domains). Thus, the current study adds to our understanding of how children’s academic, artistic, and athletic competencies are perceived by different raters and across different domains.

The first specific aim of the current study was to evaluate both correlational and mean level similarities and differences in ratings of perceptions of children’s math, reading, music, and sports competencies according to children, mothers, fathers, and teachers. Consistent with the broader literature reviewed above, for inter-rater comparisons (Hypothesis 1a), we hypothesized higher agreement (i.e., larger correlations and fewer mean level differences) regarding children’s competencies between mothers and fathers than between the other pairs of reporters. We also hypothesized that teachers’ perceptions of children’s competencies would be the most different from the other reporters. Furthermore, we hypothesized that teachers would provide the highest mean competence ratings out of the four reporters. Based on literature highlighting differences in reports of children’s functioning according to the domain assessed (De Los Reyes et al., 2015), we also hypothesized about several inter-domain comparisons among math, reading, music, and sports competencies (Hypothesis 1b). Specifically, we hypothesized higher agreement (i.e., larger correlations and fewer mean differences) regarding reports of children’s competencies in math and reading than those for music and sports, given that performance in these academic domains is more easily observable through homework and test grades. This hypothesis is supported by literature indicating that children are viewed as more competent in core academic courses as compared to extracurricular activities (Wigfield et al., 1997; Jacobs et al., 2002).

Our second specific aim was to compare patterns of inter-reporter agreement and disagreement about children’s competencies across all four domains (Hypothesis 2a) and patterns of inter-domain agreement and disagreement across all four reporters (Hypothesis 2b). Most previous studies have evaluated reporter agreement at the bivariate level, which may mask more nuanced variations in ratings of children’s competencies. For instance, it may be that correlation matrices of math and reading differ, suggesting potential differences in the overall degree of reporter agreement depending on the competence domain assessed. We therefore compared correlation matrices of child, mother, father, and teacher reports of children’s competencies in math, reading, music, and sports across reporters and domains (e.g., comparing the matrix of correlations of children’s competencies according to mother report to the matrix of correlations according to teacher report; comparing the correlation matrix of children’s math competencies to the matrix of correlations of children’s reading competencies). Given the novelty of these analyses, we did not have any specific directional hypotheses for this study goal.

## Materials and Methods

### Participants

European American families with healthy first-born and second-born children were recruited through newspaper advertisements and mass mailings from the mid-Atlantic region of the United States. The current analyses focus on data collected from children, mothers, fathers, and teachers when children were 10 years old. There were no competence data available for 84 (23.93%) of the 351 families who contributed data to the 10-year assessment. We therefore only analyzed data from families where at least one reporter provided information regarding children’s competencies in at least one of the four domains (i.e., math, reading, music, and sports), yielding a total sample size of 267 for the current study. Children were on average 10.27 years of age (*SD* = 0.18, *range* = 9.76 – 10.90) and approximately half (*n* = 136; 50.94%) were boys. Mothers were on average 41.33 years old (*SD* = 5.18, *range* = 26.94 – 55.87), and fathers were 43.58 years (*SD* = 6.41, *range* = 27.78 – 67.93). The majority of the mothers and fathers were married and living together (*n* = 214; 80.15%) and had obtained at least a bachelor’s degree (mothers: 67.79%, *n* = 181; fathers: 63.67%, *n* = 170). Families were on average from a middle to upper socioeconomic status (SES) on the Hollingshead (1975) Four-Factor Index of Social Status, and ranged from lower to upper socioeconomic status (*M* = 54.77, *SD* = 9.90, *range* = 25 – 66). Of the teachers who provided data to the 10-year assessment, the majority were 4th (37.45%, *n* = 100) or 5th (27.72%, *n* = 74) grade teachers (6 [2.25%] from combined 3rd–4th or 4th–5th grade classrooms; 87 [32.6%] missing/no response). Written informed consent was obtained from the parents of child participants and from parents and teachers for their participation.

### Procedure

Measures collected for the current study were part of a larger assessment battery that included home and laboratory visits. Children’s competencies were assessed at the home visit. Packets of questionnaires were mailed to mothers and fathers prior to their visits. Mothers were also asked to provide their children’s teachers with a packet of questionnaires to complete. The packet contained a letter explaining the study, a consent form, and a self-addressed stamped envelope to return the completed questionnaires directly to the research team. Informed consent was obtained from mothers, fathers, and teachers, and assent was obtained from children. Study procedures were approved and monitored by our Institutional Review Board.

### Measures

#### Children’s Competencies

Children, mothers, fathers, and teachers each completed the Children’s Competence Beliefs and Subjective Task Values (CBTV; Eccles et al., 1993; Eccles and Wigfield, 1995; Wigfield et al., 1997) measure to assess individual perceptions of children’s competencies in the domains of math, reading, music, and sports. Reporters are asked to rate children’s competencies in these domains on a 7-point rating scale, with higher scores indicating better competence. Subscale scores used in the current study were based on a factor analysis conducted by Wigfield et al. (1997). Items used to assess children’s, mothers’, fathers’, and teachers’ perceptions of children’s competencies in math, reading, music, and sports are included in the Appendix. Subscale scores were calculated by taking the mean of the items within each domain according to each reporter. Summary means across raters and domains were calculated by taking the average of these subscales (e.g., the child-report summary mean was calculated by taking the average of the child-reported math, reading, music, and sports subscales). The subscales used in the current study demonstrated good reliability according to child (α_*Math*_ = 0.86, α_*Reading*_ = 0.86, α_*Music*_ = 0.76, α_*Sports*_ = 0.91), mother (α_*Math*_ = 0.90, α_*Reading*_ = 0.92, α_*Music*_ = 0.89, α_*Sports*_ = 0.92), father (α_*Math*_ = 0.93, α_*Reading*_ = 0.93, α_*Music*_ = 0.91, α_*Sports*_ = 0.93), and teacher (α_*Math*_ = 0.89, α_*Reading*_ = 0.89, α_*Music*_ = 0.81, α_*Sports*_ = 0.89) reports. In keeping with our current focus on perceptions of children’s academic competencies, we only examined items relevant to children’s competencies and not items related to subjective value reports.

### Analysis Plan

Analyses were conducted in SPSS 21 (for Hypothesis 1) and *Mplus* 7.2 (for Hypothesis 2; Muthén and Muthén, 2014). To account for the nested structure of the data from families where two children participated (i.e., first- and second-borns), we clustered based on family and used the Huber-White adjustment of the standard errors to account for non-independence. We used a maximum likelihood estimator that calculated robust standard errors (MLR; Little and Rubin, 2002). Missing data were due to non-response of one or more family members and to non-response to individual items. Specifically, data regarding 10-year competencies were available from all 267 children, 225 (84.27%) mothers, 202 (75.66%) fathers, and 198 (74.16%) teachers. Missing data were handled with full-information maximum likelihood (FIML) in *Mplus*. Data were missing completely at random, as evidenced by a non-significant Little’s MCAR test, χ*^2^*(336) = 338.10, *p* = 0.458.

For our first aim investigating mean and correlational similarities and differences in reports of children’s competencies, we first examined inter-rater and inter-domain bivariate correlations to examine degrees of agreement in ratings of children’s competencies in math, reading, music, and sports. Specifically, we tested for differences in dependent inter-rater and inter-domain correlations via a web utility developed by Lee and Preacher (2013) based on methods identified by Steiger (1980). We then conducted a series of paired-samples *t*-tests to determine if, at the bivariate level, the mean-level ratings of children’s competencies were different among individual reporters and domains. We also examined if the summary means differed across reporters and domains.

For our second aim comparing patterns of inter-reporter and inter-domain agreement about children’s competencies, all competence variables were standardized prior to analyses to aid in interpretation of the coefficients. We first estimated two correlation matrices (e.g., the correlation matrices of children’s math and reading competencies) simultaneously and allowed all bivariate correlations within those matrices to be freely estimated (i.e., an unconstrained, fully free model). We then constrained all paired bivariate associations within the correlation matrices to be equal to each other (i.e., a fully constrained model). For instance, when comparing the correlation matrices of math and reading, we constrained the correlation of child and mother reports of math to be equal to the correlation of child and mother reports of reading, and so on for all six paired correlations (refer to Table 1). If this fully constrained model provided a significantly worse fit to the data than the unconstrained model, we examined modification indices and freed constraints with the highest modification indices (Yoon and Kim, 2014). Individual constraints were therefore freed progressively, until the nested model test reached non-significance (i.e., a partially constrained model). Nested model tests (comparing the constrained versus unconstrained models) were conducted via the Satorra-Bentler scaled Δχ*^2^*-difference test (Satorra and Bentler, 2001). Non-significant Δχ*^2^*-difference tests indicated that a more restrictive model (i.e., the model with more parameter equality constraints) did not provide a significantly worse fit to the data than a less restrictive model (i.e., the model with fewer parameter constraints).

**TABLE 1:** Means, standard deviations, and inter-rater correlations of child math, reading, music, and sports competencies.

	**A *Math Competence***				**B *Reading Competence***				**C *Music Competence***				**D *Sports Competence***				**Mean (*SD*)**
**Variable**	***1***	***2***	***3***	***4***	***1***	***2***	***3***	***4***	***1***	***2***	***3***	***4***	***1***	***2***	***3***	***4***	
(1) Child	–	0.57^∗∗∗^	0.48^∗∗∗^	0.36^∗∗∗^	–	0.63^∗∗∗^	0.55^∗∗∗^	0.40^∗∗∗^	–	0.45^∗∗∗^	0.49^∗∗∗^	0.38^∗∗∗^	–	0.67^∗∗∗^	0.65^∗∗∗^	0.36^∗∗∗^	5.26 (0.67)
(2) Mother	0.68	–	0.70^∗∗∗^	0.48^∗∗∗^	0.60	–	0.78^∗∗∗^	0.52^∗∗∗^	0.80	–	0.53^∗∗∗^	0.24^∗∗^	0.55	–	0.74^∗∗∗^	0.43^∗∗∗^	5.27 (0.74)
(3) Father	0.77	0.51	–	0.40^∗∗∗^	0.70	0.39	–	0.53^∗∗∗^	0.76	0.72	–	0.30^∗∗∗^	0.58	0.45	–	0.53^∗∗∗^	5.19 (0.75)
(4) Teacher	0.87	0.77	0.84	–	0.84	0.73	0.72	–	0.86	0.94	0.91	–	0.87	0.82	0.72	–	5.18 (1.04)
Mean (*SD*)	5.34 (1.08)	5.70 (1.09)	5.67 (1.10)	5.55 (1.19)	5.57 (1.02)	5.74 (1.24)	5.73 (1.25)	5.46 (1.20)	4.80 (1.29)	4.69 (1.35)	4.66 (1.26)	4.60 (1.32)	5.38 (1.30)	4.91 (1.40)	4.71 (1.33)	4.95 (1.57)	

*Numbers above the diagonal in matrices are correlations; numbers below the diagonal indicate proportions of unexplained variance between the two variables. Possible scores on the CBTV range from 1 to 7. ^∗∗^*p* < 0.01. ^∗∗∗^*p* < 0.001.*

## Results

### Hypothesis 1: Rank-Order and Mean-Level Similarities and Differences in Ratings of Children’s Competencies

#### Hypothesis 1a: Inter-Rater Comparisons

Correlations in Table 1 (above the diagonal) indicate significant positive associations between all reporters, such that higher ratings of children’s competencies according to one reporter are related to higher competence ratings according to the other reporters. Testing for differences in these dependent correlations revealed that mothers and fathers tended to agree with each other more than the other reporters for all four domains (all *z*s > 2.43; all *p*s < 0.05), except when compared to child–mother (*z* = 1.77, *p* = 0.076) and child–father (*z* = 0.87, *p* = 0.384) agreement in music competency. Children and mothers tended to agree more than children and fathers on math (*z* = 2.63, *p* = 0.008) and reading (*z* = 2.89, *p* = 0.004) competencies; more than children and teachers on math (*z* = 4.57, *p* < 0.001), reading (*z* = 5.47, *p* < 0.001), and sports (*z* = 6.93, *p* < 0.001) competencies; and more than mothers and teachers on reading (*z* = 2.50, *p* = 0.013), music (*z* = 3.87, *p* < 0.001), and sports (*z* = 5.23, *p* < 0.001) competencies. Children and fathers also tended to agree more than children and teachers on competencies in all four domains (all *z*s > 2.02; all *p*s < 0.05), and more than fathers and teachers on music (*z* = 3.60, *p* < 0.001) and sports (*z* = 2.71, *p* = 0.007) competencies. Mothers and teachers agreed more than children and teachers on math (*z* = 2.74, *p* = 0.006) and reading (*z* = 3.02, *p* = 0.002) competencies, and children and teachers agreed more than mothers and teachers on music competency (*z* = 2.67, *p* = 0.008). Mothers and teachers also agreed more than fathers and teachers on math competency (*z* = 2.19, *p* = 0.028), and fathers and teachers agreed more than children and teachers on reading competency (*z* = 3.00, *p* = 0.003). Fathers and teachers also agreed more than children and teachers (*z* = 4.38, *p* < 0.001) and mothers and teachers (*z* = 3.02, *p* = 0.003) on sports competency. Hence, as hypothesized, mothers and fathers tended to have the strongest agreement and teachers tended to have lower agreement with all other reporters.

Overall tests of mean-level inter-rater agreement (aggregating across domains) indicated that children, mothers, fathers, and teachers provided similar ratings of children’s competencies.

However, within-domain paired-samples *t-*tests indicated some degree of systematic inter-reporter disagreement (see Table 2), such that children and mothers rated children’s math, reading, and sports competencies differently (children rated themselves lower than mothers in math and reading but higher in sports competencies). Children and fathers rated children’s math, music, and sports competencies differently (children rated themselves lower than fathers in math but higher in music and sports competencies). Children and teachers and mothers and fathers only differed in ratings of sports competence, with children rating themselves higher than teachers and mothers providing higher ratings than fathers. Mothers also provided higher ratings of children’s math and reading competencies compared to teachers, and fathers provided higher ratings of children’s reading competencies compared to teachers.

**TABLE 2:** Average inter-rater difference scores of child math, reading, music, and sports competencies.

	***Math Competence***			***Reading Competence***			***Music Competence***			***Sports Competence***			***Overall (Across Domains)***		
**Variable**	**Mean (*SD*)**	***t* (*df*)**	***d***	**Mean (*SD*)**	***t* (*df*)**	***d***	**Mean (*SD*)**	***t* (*df*)**	***d***	**Mean (*SD*)**	***t* (*df*)**	***d***	**Mean**	***t* (*df*)**	***d***
Children vs. mothers	−0.35 (0.99)	−5.34 (223)^∗∗∗^	−0.33	−0.16 (0.98)	−2.50 (215)^∗^	−0.15	0.13 (1.39)	1.35 (218)	0.10	0.46 (1.10)	6.13 (214)^∗∗∗^	0.34	−0.01	−0.14 (223)	−0.01
Children vs. fathers	−0.34 (1.11)	−4.32 (197)^∗∗∗^	−0.32	−0.15 (1.11)	−1.91 (195)	−0.13	0.21 (1.29)	2.31 (197)^∗^	0.17	0.67 (1.10)	8.49 (194)^∗∗∗^	0.51	0.07	1.56 (199)	0.12
Children vs. teachers	−0.14 (1.27)	−1.53 (192)	−0.12	0.09 (1.22)	1.03 (187)	0.08	0.19 (1.47)	1.67 (174)	0.14	0.51 (1.63)	4.16 (177)^∗∗∗^	0.35	0.08	1.40 (194)	0.12
Mothers vs. fathers	0.04 (0.87)	0.65 (180)	0.04	0.03 (0.82)	0.45 (181)	0.02	−0.06 (1.23)	−0.62 (178)	−0.05	0.16 (1.00)	2.19 (180)^∗^	0.12	0.08	0.89 (181)	0.06
Mothers vs. teachers	0.24 (1.14)	2.81 (170)^∗∗^	0.22	0.30 (1.16)	3.42 (171)^∗∗^	0.26	0.04 (1.67)	0.32 (153)	0.03	0.07 (1.57)	0.54 (162)	0.05	0.09	1.65 (172)	0.14
Fathers vs. teachers	0.16 (1.05)	1.05 (152)	0.09	0.22 (1.16)	2.39 (154)^∗^	0.19	0.07 (1.54)	0.51 (141)	0.05	−0.13 (1.45)	−1.11 (147)	−0.09	0.01	0.12 (155)	0.01

**t*-statistics indicate if the means are significantly different from each other (paired-samples *t*-test). ^∗^*p* < 0.05. ^∗∗^*p* < 0.01. ^∗∗∗^*p* < 0.001.*

#### Hypothesis 1b: Inter-Domain Comparisons

Compared to the inter-rater comparisons as hypothesized, fewer significant correlations (66.67%) obtained between domains (see Table 3). Specifically, only ratings of children’s reading and music competencies were associated according to child report. According to mother and father reports, children’s math and reading competencies, math and sports competencies, and reading and music competencies were positively related. However, the association between children’s reading and sports competencies was negative according to mother report, indicating that mothers viewed more competence in reading as related to less competence in sports. According to fathers’ reports, associations between children’s math and music competencies, and music and sports competencies, were positive. All correlations according to teacher reports were positive, indicating that teachers perceived more child competence in one domain as related to more competence in other domains.

**TABLE 3:** Means, standard deviations, and inter-domain correlations of child math, reading, music, and sports competencies according to child, mother, father, and teacher reports.

	**A *Child Report***				**B *Mother Report***				**C *Father Report***				**D *Teacher Report***				**Across-rater Mean (*SD*)**
**Variable**	***1***	***2***	***3***	***4***	***1***	***2***	***3***	***4***	***1***	***2***	***3***	***4***	***1***	***2***	***3***	***4***	
(1) Math	–	0.09	0.01	0.12	–	0.40^∗∗∗^	0.13	0.14^∗^	–	0.42^∗∗∗^	0.16^∗^	0.15^∗^	–	0.68^∗∗∗^	0.50^∗∗∗^	0.43^∗∗∗^	5.51 (0.92)
(2) Reading	0.99	–	0.31^∗∗∗^	-0.10	0.84	–	0.17^∗^	−0.14^∗^	0.82	–	0.18^∗^	-0.08	0.54	–	0.53^∗∗∗^	0.24^∗∗^	5.60 (1.03)
(3) Music	1.00	0.90	–	0.09	0.98	0.97	–	0.02	0.97	0.97	–	0.18^∗^	0.75	0.72	–	0.43^∗∗∗^	4.68 (1.02)
(4) Sports	0.99	0.99	0.99	–	0.98	0.98	1.00	–	0.98	0.99	0.97	–	0.82	0.94	0.82	–	5.02 (1.18)
Mean (*SD*)	5.34 (1.08)	5.57 (1.02)	4.80 (1.29)	5.38 (1.30)	5.70 (1.09)	5.74 (1.24)	4.69 (1.35)	4.91 (1.40)	5.67 (1.10)	5.73 (1.25)	4.66 (1.26)	4.71 (1.33)	5.55 (1.19)	5.46 (1.20)	4.60 (1.32)	4.95 (1.57)	

*Numbers above the diagonal in matrices are correlations; numbers below the diagonal indicate proportions of unexplained variance between the two variables. Possible scores on the CBTV range from 1 to 7. ^∗^*p* < 0.05. ^∗∗^*p* < 0.01. ^∗∗∗^*p* < 0.001.*

Testing for differences in these dependent correlations indicated that mothers, fathers, and teachers all viewed children’s math and reading competencies as more strongly related with each other than relations among the other domains (all *z*s > 3.45; all *p*s < 0.01). Children viewed their reading and music competencies as more strongly correlated with each other than relations among the other domains (all *z*s > 2.88; all *p*s < 0.01), and mothers, fathers, and teachers perceived children’s reading and music competencies as more strongly related than their reading and sports competencies (all *z*s > 3.84; all *p*s < 0.001). All reporters viewed music and sports competencies as more highly related than reading and sports competencies (all *z*s > 2.33; all *p*s < 0.05). Last, mothers, fathers, and teachers viewed child math and sports competencies as more closely related than reading and sports competencies (all *z*s > 4.02; all *p*s < 0.001), and children saw their reading and sports competencies as more highly associated than their math and sports competencies (*z* = 3.07, *p* = 0.002).

Consistent with the lower inter-domain correlations, paired-samples *t-*tests indicated more systematic inter-domain disagreement (see Table 4) than was observed among raters. Overall tests of mean-level inter-domain agreement (aggregating across reporters) indicated that ratings of children’s math competence were not significantly different from overall ratings of reading competence, but that overall math and reading competencies were rated higher than music and sports. Overall ratings of children’s sports competence were also higher than overall ratings of children’s music competence. In within-reporter paired *t*-tests, competency ratings of math compared to reading were different only according to child-report (with reading rated higher than math); however, children’s math and reading competencies were consistently rated higher than their music competency according to all four reporters. Children’s math and reading competencies were rated as higher than their sports competency according to mothers, fathers, and teachers, whereas children’s sports competency was rated higher than their music competency according to children and teachers.

**TABLE 4:** Average inter-domain difference scores of child math, reading, music, and sports competencies.

	***Child Report***			***Mother Report***			***Father Report***			***Teacher Report***			***Overall (Across Reporters)***		
**Variable**	**Mean (*SD*)**	***t* (*df*)**	***d***	**Mean (*SD*)**	***t* (*df*)**	***d***	**Mean (*SD*)**	***t* (*df*)**	***d***	**Mean (*SD*)**	***t* (*df)***	***d***	**Mean**	***t* (*df)***	***d***
Math vs. reading	−0.22 (1.42)	−2.45 (248)^∗^	−0.21	−0.04 (1.28)	−0.43 (224)	−0.03	−0.07 (1.27)	−0.72 (200)	−0.06	0.11 (0.96)	1.56 (193)	0.09	−0.09	−1.10 (263)	−0.07
Math vs. music	0.54 (1.68)	5.22 (260)^∗∗∗^	0.46	1.02 (1.62)	9.31 (220)^∗∗∗^	0.83	1.01 (1.53)	9.38 (200)^∗∗∗^	0.86	0.91 (1.26)	9.64 (177)^∗∗∗^	0.73	0.83	10.54 (266)^∗∗∗^	0.86
Math vs. sports	−0.04 (1.59)	−0.37 (247)	−0.03	0.79 (1.65)	7.22 (224)^∗∗∗^	0.63	0.96 (1.59)	8.51 (199)^∗∗∗^	0.79	0.61 (1.51)	5.55 (185)^∗∗∗^	0.44	0.49	5.97 (263)^∗∗∗^	0.47
Reading vs. music	0.74 (1.37)	8.52 (248)^∗∗∗^	0.64	1.06 (1.68)	9.41 (220)^∗∗∗^	0.82	1.07 (1.61)	9.49 (201)^∗∗∗^	0.86	0.80 (1.23)	8.72 (177)^∗∗∗^	0.64	0.92	12.27 (263)^∗∗∗^	0.90
Reading vs. sports	0.19 (1.74)	1.71 (248)	0.16	0.83 (1.99)	6.24 (224)^∗∗∗^	0.63	1.02 (1.89)	7.63 (200)^∗∗∗^	0.79	0.52 (1.73)	4.08 (184)^∗∗∗^	0.37	0.58	5.55 (263)^∗∗∗^	0.52
Music vs. sports	−0.55 (1.75)	−4.94 (247)^∗∗∗^	−0.42	−0.19 (1.92)	−1.50 (220)	−0.14	−0.04 (1.65)	−0.36 (200)	−0.03	−0.29 (1.56)	−2.49 (175)^∗^	−0.20	−0.34	−3.71 (263)^∗∗∗^	−0.31

**t*-statistics indicate if the means are significantly different from each other (paired-samples *t*-test). ^∗^*p* < 0.05. ^∗∗^*p* < 0.01. ^∗∗∗^*p* < 0.001.*

### Hypothesis 2: Comparing Patterns of Agreement and Disagreement in Ratings of Children’s Competencies

#### Hypothesis 2a: Inter-Rater Patterns Across All Four Domains

Comparing the matrices of domain correlations (e.g., comparing panels A, B, C, and D in Table 3) revealed differences in inter-rater patterns of agreement. Only the fully constrained model comparing mother and father intercorrelations did not provide a significantly worse fit to the data than the fully free (i.e., unconstrained) model, Δχ*^2^*(6) = 4.04, *p* = 0.671. This finding points to global similarities in the associations between mother and father reports of children’s math, reading, music, and sports competencies. The fully constrained models comparing inter-domain correlation matrices for child and mother, child and father, child and teacher, mother and teacher, and father and teacher reports provided a significantly worse fit to the data than the fully free models, Δχ*^2^_*child–mother*_*(6) = 22.43, *p* = 0.001; Δχ*^2^_*child–father*_*(6) = 20.71, *p* = 0.002; Δχ*^2^_*child–teacher*_*(6) = 51.67, *p* < 0.001; Δχ*^2^_*mother–teacher*_*(6) = 36.18, *p* < 0.001; Δχ*^2^_*father–teacher*_*(6) = 19.50, *p* = 0.003. Constraints were progressively freed until the nested model test reached non-significance.

The partially constrained models comparing child and mother, and child and father, reports did not provide a significantly worse fit to the data than the fully free models, Δχ*^2^_*child–mother*_*(5) = 8.64, *p* = 0.124; Δχ*^2^_*child–father*_*(5) = 7.78, *p* = 0.169. In these models the associations between math and reading competencies were freed, and all other inter-domain associations were constrained to be equal, indicating similarities in the associations among these domains according to child and mother, and child and father, reports. However, the association between child-reported math competence and child-reported reading competence (*r* = 0.10) was lower than the associations between mother- and father-reported math competence and mother- and father-reported reading competence (both *r*s = 0.40; both *z*s = −3.71, *p* < 0.001).

Nested model tests did not reach non-significance after progressively freeing all constraints for the models comparing child and teacher, mother and teacher, and father and teacher reports, and therefore for these comparisons the fully free models provided the best fit to the data. All inter-domain associations were freely estimated, indicating global differences in ratings of children’s math, reading, music, and sports competencies between these reporters. Specifically, child-, mother-, and father-reported associations were consistently lower (*r*s_*child*_ ranged from −0.10 to 0.31; *r*s_*mother*_ ranged from −0.14 to 0.40; *r*s_*father*_ ranged from −0.08 to 0.41) than teacher-reported associations (*r*s ranged from 0.25 to 0.68) across all domains (there is some overlap in these ranges, but the individual comparisons between child, mother, and father associations were all lower than the teacher associations; see Table 3; all *z*s < −3.86, *p*s < 0.001).

#### Hypothesis 2b: Inter-Domain Patterns Across All Four Raters

Comparing the matrices of reporter correlations (e.g., comparing panels A, B, C, and D in Table 1) revealed fewer differences in inter-domain patterns of agreement compared to the inter-rater patterns. The fully constrained models comparing ratings of children’s competencies in math and reading, math and music, math and sports, reading and sports, and music and sports did not provide a significantly worse fit to the data than the fully free (i.e., unconstrained) models, Δχ*^2^_*math–reading*_*(6) = 2.82, *p* = 0.831; Δχ*^2^_*math*__–__*music*_*(6) = 9.95, *p* = 0.127; Δχ*^2^_*math*__–__*sports*_*(6) = 6.18, *p* = 0.403; Δχ*^2^_*reading–sports*_*(6) = 4.17, *p* = 0.653; Δχ*^2^_*music*__–__*sports*_*(6) = 11.29, *p* = 0.080. These findings indicated similarities in the associations between math and reading, math and music, math and sports, reading and sports, and music and sports across all four reporters (e.g., the correlation between child-reported and mother-reported math was similar to the correlation between child-reported and mother-reported reading).

The fully constrained model comparing ratings of children’s reading and music competence provided a significantly worse fit to the data than the fully free model, Δχ*^2^*(6) = 14.84, *p* = 0.021. Constraints were progressively freed until the nested model test reached non-significance. The partially constrained model did not provide a worse fit to the data than the fully free model, Δχ*^2^*(3) = 6.35, *p* = 0.096. For the reading-music comparison, the associations between children and mothers, mothers and teachers, and fathers and teachers were progressively released and freely estimated. Examination of the correlation coefficients indicated that the associations between child and mother, mother and teacher, and father and teacher reports for reading competence were higher (*r*_*child*__–__*mother*_ = 0.64; *r*_*mother*__–__*teacher*_ = 0.50; *r*_*father*__–__*teacher*_ = 0.52) than those associations for music competence (*r*_*child*__–__*mother*_ = 0.47; *r*_*mother*__–__*teacher*_ = 0.25; *r*_*father*__–__*teacher*_ = 0.29), respectively (*z*_*child*__–__*mother*_ = 2.85, *p* = 0.004; *z*_*mother*__–__*teacher*_ = 3.38, *p* = 0.001; *z*_*father*__–__*teacher*_ = 3.19, *p* = 0.001). Associations between ratings from children and fathers, children and teachers, and mothers and fathers were constrained to be equal across reading and music competencies, indicating similarity in these associations.

## Discussion

The current study examined child, mother, father, and teacher perceptions of children’s competencies in math, reading, music, and sports and utilized two revealing analytic approaches to evaluate inter-rater and inter-domain agreement, rank order and mean level. In doing so, this study extends previous findings with Expectancy-Value Theory (Eccles and Wigfield, 1995; Wigfield and Eccles, 2000) and supports the need to consider perceptions of the self and other socializers across multiple domains when conceptualizing children’s overall academic, artistic, and athletic functioning. EVT posits that children’s academic performance and behavior are influenced by relations among beliefs about how well children will complete an academic task and how much value is placed on those tasks. Several academic constructs are conceptualized in this theoretical model, including children’s interests, affect, values, choices, effort, task difficulty, and *competence*. EVT proposes that perceptions of children’s academic competencies constitute a first step in this process, whereby these views influence how much a child will value and express interest in an academic task. It stands to reason then that children who do not perceive themselves as having high levels of academic competence will subsequently demonstrate little interest and see little value in academic tasks. Other studies with EVT document that low levels of academic interest, expectancies, and subjective task value lead to low academic performance and outcomes (Wigfield, 1994; Eccles and Wigfield, 1995; Wigfield et al., 1997; Simpkins et al., 2012; Trautwein et al., 2012) as well as poor career attainment (Lauermann et al., 2017). As such, initial perceptions of children’s academic competencies may have far-reaching effects on their later academic and educational success.

Our results suggest that academic perceptions of the self and other socializers tend to vary systematically. Extending the literature on EVT, perceptions of academic competence from other socializers are also important to consider, such that if children assume their parents or teachers perceive their competency in a certain domain to be relatively low, children may subsequently “live up” to those expectations (Jussim and Harber, 2005; Tomasetto et al., 2015). Perceptions of children’s academic competencies from other socializers may also “spill over” into children’s own views and expectations of how much they should value certain academic domains. For instance, if a child’s parent perceives the child to have high levels of math competency but low levels of reading competency, the child may place more value and effort in math and less in reading. The child may then eventually develop difficulties in reading as a result of those perceptions. Discrepancies in perceptions are also important to consider. That is, perceptions of high levels of math competency according to the child, but low levels of math competency according to the parent, may create conflict in expectations and values, which may in turn negatively impact children’s academic performance. Borrowing from the broader reporter discrepancy literature, the most negative outcomes are observed when two reporters harbor differing viewpoints on a child’s functioning and behavior (De Los Reyes et al., 2015). Similar findings may be observed when applied to children’s academic competencies and the broader EVT model.

Clearly, understanding how to incorporate inter-reporter and inter-domain discrepancies into theoretical models of children’s academic competencies is a crucial direction for future research. More work is also needed to understand how these differences in perceptions relate to social cognitive factors that influence academic performance. Models examining EVT should therefore consider the potential for discrepancies in reports of children’s academic competencies across raters and domains, and several methodological advances have provided the statistical modeling techniques necessary for such investigations (e.g., polynomial regression, multitrait-multimethod models, latent interaction models; Jager et al., 2012; Trautwein et al., 2012; Laird and De Los Reyes, 2013).

### Are There Differences in How Children’s Competencies Are Perceived by Different Raters?

Findings suggested a relatively high level of agreement across reporters’ ratings of children’s competencies within the four domains assessed in the current study. The correlational findings coincided with our results examining difference scores, such that there were no significant overall differences among child, mother, father, and teacher reports of children’s competencies. This documented agreement among reporters’ ratings of children’s competencies may be attributable to the observable nature of children’s abilities. For instance, homework assignments, instructor/coach feedback, test performance, and grades on report cards may provide children, parents, and teachers with a consistent sense of how children perform in a given domain.

However, testing for differences between dependent correlations revealed a more nuanced picture of cross-reporter perceptions of children’s diverse competencies. Consistent with our hypotheses, as well as the extant literature (Schroeder et al., 2010; De Los Reyes et al., 2015), mothers and fathers tended to demonstrate the most agreement in perceptions of children’s competencies. Generally higher levels of agreement were also noted between children and mothers, and mothers and teachers, as compared to other reporter dyads, perhaps reflecting mothers’ stereotypically greater involvement in and knowledge of their children’s performance and functioning (Grolnick and Slowiaczek, 1994). Additionally, agreement between teachers and other reporters tended to be lower in several domains, perhaps reflecting teachers’ unique perspectives of children’s academic functioning. Notably, we observed lower agreement between children and teachers, as compared to mothers and teachers and fathers and teachers, perhaps reflecting home-school communication between parents and teachers through parent–teacher conferences and report cards of which children in the elementary school years may be mostly unaware.

The mean differences on about half of the inter-rater comparisons also point to some systematic differences in how children, mothers, fathers, and teachers rate children’s competencies, but they were not consistent across domains (as indicated by non-significant global *t*-tests). No differences were noted between mothers’ and fathers’, and children’s and teachers’, average ratings of child competency in any domain except sports. Furthermore, no difference was noted in average ratings of math and reading competencies between children and teachers, but mothers tended to rate these competencies higher than children and teachers. Despite child–teacher agreement in mean level, child–teacher correlations tended to be lower than child–mother and mother–teacher correlations. Hence, the rank-order alignment of children and teachers was less consistent (relative to child–mother and mother–teacher alignment), but their overall assessments of competence in the group were similar.

Whereas the bivariate correlations and overall *t*-tests pointed to relatively high levels of agreement across all four reporters, analyses comparing the correlation matrices provided a further differentiated picture of degrees of reporter agreement. Specifically, child-reported math-reading associations were lower than those according to both mother and father reports, suggesting that parents perceive stronger relations between children’s competencies in these two domains than do children. Differences also emerged between teacher-reported associations and those reported by the other three reporters, such that all teacher-reported associations were higher than those reported by children and by parents. These strong correlations may be attributable to teachers having a unique perspective on children’s school functioning, as they may see more consistencies in children’s competencies across domains than do children or parents who are normally limited to seeing individual children’s grades on report cards. This finding may also reflect the fact that teachers may form a more global view of children’s competencies that generalizes across domains (Bornstein and Putnick, 2019), whereas children and parents may see more differentiated competencies. Specifically, core classroom teachers may have less information about children’s performance in music and sports given that they do not generally teach those subjects to their students. Teachers may therefore not observe students in these settings, perhaps leading teachers to generalize or stereotype children’s competencies in those areas based on children’s performance in core academic subjects. These correlations may also be due to biases in teachers’ perceptions of children’s competencies (den Brok et al., 2004), as it may be that a teacher who views a student as struggling in one domain assumes that the student is having similar difficulties in other domains. It is therefore important to consider the influence of teachers’ undifferentiated expectations and perceptions of children’s competencies.

This set of findings has clear educational implications, suggesting that teacher input should be carefully considered when discussing children’s academic functioning. The findings also suggest that many procedures already utilized in schools (e.g., midterm reports, parent–teacher conferences) provide parents with information regarding children’s academic competencies, which may in turn decrease potential areas of conflict between parents and teachers. Likewise, it may be important to include children in some of these information procedures to provide children with additional perspectives about how they are functioning in the educational setting. Such discussions may provide children with developmentally appropriate feedback on strengths and weaknesses and motivation to improve in any needed areas. Incorporating parents’ perceptions of their children’s academic competencies in broader, standard educational assessments may also help calibrate expectations for children’s academic performance, highlight academic strengths and weaknesses, and suggest potential educational interventions to support children’s academic success.

### Are There Differences in How Children’s Competencies Are Perceived Across Different Domains?

Inter-domain correlations tended to be smaller and less consistent than inter-rater correlations, and significant inter-domain disagreement emerged in the difference scores of children’s competencies of math, reading, music, and sports. Taken together, these results were consistent with our hypotheses, as well as the broader literature (Jacobs et al., 2002), that children would be perceived as more competent in core academic subjects (e.g., math and reading) than in extracurricular ones (e.g., music and sports). It is important to note consistent agreement between ratings of children’s math and reading competencies (except according to child report), supporting the strong links between math and reading abilities as well as the common cognitive skills underlying these competencies (Hart et al., 2009; Bornstein and Putnick, 2019). Additionally, ratings of children’s music competence were lower than ratings for their math, reading, and sports competencies. This finding may reflect a general lack of exposure to and practice with musical instruments in elementary schools in the United States (Wigfield et al., 1997). The lower ratings of children’s music competence may also reflect the fact that fewer questions assessing music competence are included in the CBTV, and therefore the music subscale may be missing some crucial aspects of children’s competencies in this domain (e.g., confidence in abilities, expectations for performance; see Appendix).

Dependent correlation tests also supported stronger associations between math and reading as compared to associations between the other domains. Stronger correlations were also revealed between music and sports, which are frequently seen as extracurricular activities, as compared to the other core academic domains. Children also consistently perceived reading and music as more strongly correlated than the other domains, and this was the only significant inter-domain correlation according to children’s reports (Table 2). Reading and music were often more highly related than other domains according to other reporters as well. This finding is supported by literature indicating a strong association between reading skills and the ability to discriminate musical sounds, as musical abilities often stem from well-developed phonemic awareness (Lamb and Gregory, 1993).

The bivariate analyses indicated differences among the four domains, but analyses comparing inter-domain correlation matrices highlighted more similarities, except between reading and music. Specifically, and consistent with the bivariate findings discussed above, associations were stronger for reading than for music competence, suggesting more consistency among reporters’ ratings of children’s reading competence than their music competence. This difference may again be attributable to the lack of exposure to music in elementary school (Wigfield et al., 1997) or to measurement differences.

It may not be surprising that more differences than similarities were noted in perceptions of children’s competencies across various domains, as children naturally demonstrate patterns of strengths and weaknesses (Bornstein and Putnick, 2019). However, the fact that more agreement was seen in core academic subjects as compared to extracurricular subjects suggests that children might require more support in and exposure to these areas. Agreement levels may also indicate that additional means of communicating about children’s competencies in special academic areas like music and sports is needed. Perhaps music teachers and sport coaches should be included in parent-teacher conferences, or other means of providing feedback to children and parents in these areas seems warranted. Taken together, the findings from the current study point to consistency in the strengths of the inter-rater associations among the four domains assessed and more variation in the inter-domain associations across the four reporters. Our results also highlight the need to consider additional methodological techniques (e.g., actual grades) to fully assess the degree of individual differences in reports of children’s academic competencies.

### Strengths, Limitations, and Future Directions

The current study has several strengths, including a multi-reporter and multi-domain design to fully evaluate differences in reporters’ perceptions of children’s elementary school competencies in several arenas, domains which to date have not been examined in the literature on reporter agreement. We also extended our analyses beyond the bivariate level, thereby providing a methodological contribution to this literature. However, we also note several limitations to the current study. Our sample is relatively sociodemographically diverse, but it is not representative of the entire range of ethnic backgrounds; therefore, these findings may not generalize to other samples (e.g., minority populations). Child development and parenting are known to vary with ethnicity (Bornstein and Lansford, 2010; Bornstein, 2015; Murry et al., 2015); by including only European American families, we intentionally avoided the ethnicity-socioeconomic status confound that has vexed the existing literature and would also cloud our findings with respect to children’s competencies and parents’ and teachers’ perceptions (Bornstein et al., 2013; Jager et al., 2017). It is also not clear if the associations and disagreements documented in the current study would be observed in at-risk or clinical populations. For instance, differences in reports of children’s competencies may be even lower for children with intellectual and cognitive disabilities, as their functioning may be less variable and more readily known to both parents and teachers due to increased academic testing and accommodations.

Additionally, our measure of children’s competencies varied by the reporter and by the domain assessed in terms of the number and content of items (see Appendix). It is possible that the correlations obtained in the current study would be even stronger if the same numbers of items and similar contents had been used, as similarity in measurement may have reduced error and variation in ratings. It will be important for future studies to consider the impact of measurement differences on the degree of documented reporter disagreement. Furthermore, we cannot confirm or assume equal intervals between response options on the CBTV. Utilization of analytic techniques that relax this assumption, including many-facet Rasch models (which also evaluate severity across reporters; Myford and Wolfe, 2003), is warranted in future studies with the CBTV. We also did not have an objective measure of children’s academic competencies in the current study. Instead, the current study focuses on patterns in perceptions of children’s competencies across reporters and domains, providing a contribution to Eccles and colleagues’ EVT model (Wigfield and Eccles, 2000) of children’s functioning in educational settings.

Last, our study is cross-sectional as ratings of children’s competencies were only included at one point in time. It is important to consider that agreement in reports of children’s competencies may change over time, particularly given that child and parent reports tend to vary greatly from each other. Research also suggests that ratings of children’s competencies in math, reading, and sports decline across elementary school and then level off or even increase across the high school years (Jacobs et al., 2002). Consideration of these developmental differences is of paramount importance to understanding the extent and impact of reporter agreement and disagreement about children’s competencies. Examination of perceptions of academic competencies during adolescence (i.e., when students are in high school) is a particularly important direction for future research, as any discrepancies in these perceptions across reporters and domains may have significant implications for adolescents’ college readiness and career trajectories.

### Implications

This study provided a novel look into perceptions of child competencies by children, mothers, fathers, and teachers. The mixed agreement found between reporters has implications for educational measurement, theory, and practice. Regarding measurement, does the high level of agreement among reporters suggest that different perspectives should be combined (as in a factor score) to make a more valid measure of child competence? Perhaps not. Mother and father reports were the most consistent in both mean level and rank order (although still not interchangeable), but we found several systematic differences between other reporters, suggesting that reporter differences are not simply a result of random error. Furthermore, recent work has shown that the unshared variance between reporters (which is usually relegated to the error term in a factor model) may be meaningfully predictive of child and dyad functioning (e.g., Jager et al., 2012). Consequently, the reporter of record should be chosen based on study goals.

Regarding theory, the moderate positive relations between perceptions of child math and reading competencies for mothers, fathers, and teachers, but not children, indicates that children may be more likely to use dimensional comparisons to inform their competence ratings about themselves, but adults (i.e., parents and teachers) ratings of children may not. Adults may be more likely to draw on a working model of general (*g*) intelligence that supports positive relations between math and reading competence (Furnham et al., 2002). A hierarchical model of intelligence, where various academic competencies load on a single general factor, has been supported in studies of child intelligence and performance (McGrew, 2009; Castejon et al., 2010; Bornstein and Putnick, 2019). Hence, adults may be more accurate reporters of child competencies than children themselves. Future research assessing the predictive validity of each reporters’ competence ratings are needed to validate this deduction.

Finally, regarding educational practice, our study suggests that success is in the eye of the beholder. Mothers and fathers tend to agree about children’s competencies across domains, but teachers and children have lower levels of agreement with one another as well as with parents. Reporters’ ratings of competence also vary by domain. Teachers, administrators, and school counselors should be aware of these differences in ratings of children’s competencies by reporter as well as domain. Assessing competence from multiple sources may provide the most well-rounded picture of child competence. Furthermore, ratings of children’s academic and school competencies may develop into inherent expectations and biases, which may alter how individuals perceive children’s long-term functioning in an academic setting. Such biased evaluations (both positive and negative, and by the self and others) are related to important long-term academic outcomes, including self-regulation, social bonding, and school achievement (Jussim and Harber, 2005; Leduc and Bouffard, 2017). Acknowledging that individual ratings of children’s academic competencies are but one piece of data that needs to be verified or calibrated with other data sources may help prevent the development of any biases or false expectations in children’s academic functioning.

## Data Availability Statement

The datasets generated for this study are available on request to the corresponding author.

## Ethics Statement

The studies involving human participants were reviewed and approved by NIH IRB. Written informed consent to participate in this study was provided by the participants’ legal guardian/next of kin.

## Author Contributions

SR reviewed the literature. DP and MB supervised the testing. DP undertook the analysis. MB and DP conceived and designed the study. All the authors wrote and reviewed the manuscript. MB and GE commented and submitted the manuscript.

## Conflict of Interest

The authors declare that the research was conducted in the absence of any commercial or financial relationships that could be construed as a potential conflict of interest.

## Appendix

**TABLE:** Items to assess child, mother, father, and teacher perceptions of children’s competencies in math, reading, music, and sports.

**Child**	**Mother/Father**	**Teacher**
**Math**		
(1) How good in math are you?	(1) How good is your child in math?	(1) Compared to other children, how hard does this child try in math?
(2) If you were to list all the students in your class from the worst to the best in math, where would you put yourself?	(2) In comparison to other children, how would you evaluate your child’s performance in math?	(2) How well is this child performing in math compared to how well you believe s/he could?
(3) Compared to most of your other school subjects, how good are you in math?	(3) Compared to other children, how much innate ability or talent does this child have in math?	(3) Compared to other children, how much innate ability or talent does this child have in math?
(4) How well do you expect to do in math this year?	(4) How well do you think your child will do in math next year?	(4) How well do you expect this child to do next year in math?
(5) How good would you be at learning something new in math?		(5) Compared to other children, to what extent does this child give up when faced with a difficult problem in math?
(6) In general, how hard is math for you? (reversed)		
**Reading**		
(1) How good in reading are you?	(1) How good is your child in reading?	(1) Compared to other children, how hard does this child try in reading?
(2) If you were to list all the students in your class from worst to best in reading, where would you put yourself?	(2) In comparison to other children, how would you evaluate your child’s performance in reading?	(2) How well is this child performing in reading compared to how well you believe s/he could?
(3) Compared to most of your other school subjects, how good are you in reading?	(3) Compared to other children, how much innate ability or talent does this child have in reading?	(3) Compared to other children, how much innate ability or talent does this child have in reading?
(4) How well do you expect to do in reading this year?	(4) How confident is your child in his/her ability to do well in reading?	(4) How well do you expect this child to do next year in reading?
(5) How good would you be at learning something new in reading?		(5) Compared to other children, to what extent does this child give up when faced with a difficult problem in reading?
(6) In general, how hard is reading for you? (reversed)		
**Music**		
(1) How good are you at music?	(1) How good is your child in music?	(1) Compared to other children, how hard does this child try in music?
(2) Compared to most of your other activities, how good would you be at playing a musical instrument?	(2) Compared to other children, how much innate ability or talent does this child have in music?	(2) Compared to other children, how much innate ability or talent does this child have in music?
(3) How good would you be at learning to play a new musical instrument?	(3) In comparison to other children, how would you evaluate your child’s performance in music?	(3) Compared to other children, to what extent does this child give up when faced with a difficult problem in music?
(4) In general, how hard would learning to play a musical instrument be for you? (reversed)		
**Sports**		
(1) How good at sports are you?	(1) How good is your child in sports?	(1) Compared to other children, how hard does this child try in sports?
(2) If you were to list all the students in your class from the worst to the best in sports, where would you put yourself?	(3) Compared to other children, how much innate ability or talent does this child have in sports?	(2) Compared to other children, how much innate ability or talent does this child have in sports?
(3) Compared to most of your other activities, how good are you at sports?	(3) In comparison to other children, how would you evaluate your child’s performance in sports?	(3) Compared to other children, to what extent does this child give up when faced with a difficult problem in sports?
(4) How well do you expect to do in your favorite sport this year?	(4) How confident is your child in his/her ability to do well in sports?	
(5) How good would you be at learning a new sport?		
(6) In general, how hard are sports for you? (reversed)		
